# Knowledge, attitudes, and practices regarding schistosomiasis infection and prevention: A mixed-methods study among endemic communities of western Uganda

**DOI:** 10.1371/journal.pntd.0010190

**Published:** 2022-02-23

**Authors:** Maxson Kenneth Anyolitho, Karolien Poels, Tine Huyse, Julius Tumusiime, Faith Mugabi, Casim Umba Tolo, Caroline Masquillier, Viola Nilah Nyakato

**Affiliations:** 1 Department of Public Health, Lira University, Lira, Uganda; 2 Department of Human Development and Relational Sciences, Mbarara University of Science and Technology, Mbarara, Uganda; 3 Department of Communication Studies, University of Antwerp, Antwerp, Belgium; 4 Department of Biology, Royal Museum for Central Africa, Tervuren, Belgium; 5 Department of Biology, Mbarara University of Science and Technology, Mbarara, Uganda; 6 Department of Sociology, University of Antwerp, Antwerp, Belgium; University of Kent, UNITED KINGDOM

## Abstract

**Introduction:**

In Uganda, schistosomiasis (re)infections have continued to remain high despite the implementation of mass drug administration and sensitization campaigns aimed at controlling the disease. This could imply that there are some barriers to the implemented preventive measures. We conducted a mixed-methods study in Kagadi and Ntoroko districts around Lake Albert to assess knowledge, attitudes, and practices regarding schistosomiasis and to explore and understand perspectives regarding the disease.

**Materials and methods:**

Semi-structured survey questionnaires were administered to 337 household adults selected through systematic random sampling. We also interviewed 12 participants and held 28 focus-group discussion sessions with 251 individuals respectively. Quantitative data was analysed using frequencies, percentages, and chi-square tests for associations, while themes and sub-themes were used to analyse qualitative data respectively.

**Findings:**

A total of 98.5%, 81.3%, and 78.5% had heard about schistosomiasis, and knew the main transmission modes and symptoms, respectively. The majority (75.8%) said avoiding contact with water was a preventative way, while 67.5% said observing signs and symptoms was a form of diagnosis. Furthermore, 98.4% and 73.4% said it was important to defecate in latrines and to avoid contact with contaminated water respectively. However, it is difficult to avoid contact with lake water because it is the only source of livelihood, especially for fisher communities. Open defecation is commonly practiced along the lake due to insufficient space and difficulties in the construction of latrines. Myths and misconceptions reported include; lake water is safe, gassing in water causes transmission, fetching water early in the morning and from deep water is safe, and feces in the lake water act as a bait for catching fish.

**Conclusions and recommendations:**

Despite adequate knowledge of schistosomiasis and a positive attitude towards its prevention, existing myths and misconceptions, coupled with persistent risky water, sanitation, and hygiene practices still pose a challenge. A more robust community-based awareness intervention using bottom-up participatory approaches, accompanied by the provision of clean and safe water sources and increasing latrine coverage, could provide lasting solutions to these barriers.

## Author summary

Schistosomiasis is one of the leading neglected tropical diseases, second to malaria in prevalence. In Uganda, more than 10 million people (25.6%) are currently infected, with prevalence in some areas as high as 90%, and more than 55% of the population is at risk. The most at-risk populations are the fishing communities and school-aged children. Despite the government’s prevention and control programs such as mass drug administration (MDA) and sensitization, infections and re-infections have continued to occur, even in areas where MDA has been implemented. Previous studies have linked this with risky behavior. However, information from western Uganda is inadequate. We conducted a mixed-methods study to assess community knowledge, attitudes, and practices, and to understand their opinions and perspectives regarding the disease. The study found that, whereas there is adequate knowledge of schistosomiasis and although there is a positive attitude toward avoiding water contact and the use of latrines, it is difficult to do so, something that could explain the persistent risky practices. The risky practices could also be compounded by myths and misconceptions surrounding the disease. These findings shall form the basis for the design and implementation of contextualized, community-based, participatory communication tools for behavior change. Community involvement in schistosomiasis prevention programs could lead to increased awareness about disease prevention and could debunk existing myths and misconceptions, thereby improving behaviors, practices, and habits, ultimately lowering infection.

## Introduction

Schistosomiasis (also known as bilharzia), is a snail-borne parasitic disease that is caused by trematode blood flukes of the genus *Schistosoma* [1]. Globally, it is one of the Neglected Tropical Diseases (NTDs) and is second to malaria in terms of prevalence. It poses serious health consequences to people’s lives. Although Uganda is not among the top five countries in Sub-Saharan Africa with the highest prevalence, it is still considered highly endemic [2]. Currently, the country has more than 10 million (25.6%) adults infected with the disease and more than 14 million (36.1%), when children aged 2–4 years are included, with some areas having a prevalence as high as 92% [3]. About 55% of the Ugandan population is at risk of infection [4,5].

In Uganda, intestinal and urogenital schistosomiasis are the two common types, with intestinal schistosomiasis being endemic in most parts of the country [5]. Common signs and symptoms include a swollen belly, blood in the feces, skin rash, fever, head, and body aches, breathing difficulties, diarrhea and constipation, liver fibrosis, intestinal ulcers, and sometimes high blood pressure, stunted growth, cognitive impairment in children, and infertility [4,6]. Diagnosis is normally through the detection of the schistosome eggs during microscopical urine and stool analysis [7].

Several socio-cultural risk factors, such as knowledge, attitude, and practices (KAP) and personal characteristics such as age, education, gender, and economic and environmental factors are found to influence schistosomiasis infection and prevention [8]. There is a link between schistosomiasis infection and the level of knowledge, attitude, and water, sanitation and hygiene (WASH) practices. It is often argued that knowledge influences a person’s attitudes and beliefs, which in turn, can influence practices thereby increasing the risk of infection [9].

Limited knowledge of transmission modes, signs and symptoms, diagnosis, and prevention, also affects efforts to control schistosomiasis [10]. A recent nationally based survey conducted in Uganda revealed that only 61.8% of respondents had ever heard of schistosomiasis, a factor associated with the high prevalence of infection [3]. Relatedly, a study on the islands of Lake Victoria in Uganda, showed that although 92.3% of the biomedical staff, 84.3% of pupils, 80.4% of teachers, and 87.3% of household heads had knowledge and awareness of the disease, limited knowledge of transmission mode was reported to be a major challenge [11]. These findings imply that variations exist in the knowledge and awareness of schistosomiasis among different geographical locations and different population groups, which could influence prevention efforts. Studies also show that community attitudes toward schistosomiasis differ from place to place. A cross-sectional study conducted on the Lake Victoria islands of Uganda found positive attitudes toward efforts to control schistosomiasis [11]. On the contrary, a study of KAPs by Muhumuza [12] revealed negative attitudes toward preventive measures. Good WASH practices are important for the prevention of schistosomiasis. The absence or lack of access to clean and safe water forces communities to use contaminated sources of water, while the absence of a proper latrine encourages open defecation [12–14]. It is also reported by scholars that, a low level of knowledge and negative attitudes tend to influence risky practices such as defecation and urination in water, swimming, bathing, washing utensils, clothes, and fishing [15].

The association between schistosomiasis infections and socioeconomic factors such as age, income levels, poverty, gender, educational levels, marital status, and ethnicity have also been studied [16–18]. Previous research revealed that poverty forces individuals to engage in risky practices and results in difficulties in accessing clean water and constructing latrines [19]. A study regarding the impact of host gender on the risk of schistosomiasis infection in Mayuge district of Uganda found that both boys and girls had similar amounts of contact with water, with no significant differences between genders in infection and re-infection [18]. Although the above study did not find any difference between gender in children with regard to water practices, there is a need to look at the implications of gender variations in knowledge, attitudes, and practices regarding schistosomiasis and not just on infection and treatment alone.

Uganda was the first country to adopt the World Health Organisation’s (WHO) recommendation of mass drug administration (MDA) with the drug praziquantel in 2003 [16,20]. The program targets high-risk communities such as those around lakes, and treats infected and non-infected children aged between 5 and 12 years [21–23]. There are also programs focusing on prevention by improving WASH practices and health education [24,25]. Despite the above efforts, evidence on the ground suggests that infections and re-infections have continued to emerge [3,26]. Studies conducted in the eastern and central part of the country, especially along Lake Victoria, suggest that limited understanding of socio-cultural factors including the KAPs regarding schistosomiasis infection and prevention inhibit effective intervention strategies [3,11,23,27]. Although Lake Albert in western Uganda falls under a moderately and highly endemic region where at least 5 out of every 10 persons are said to be infected with schistosomiasis [3,28], there is a paucity of literature on community KAP regarding the disease. Based on this background, we set out to assess the current level of KAP regarding schistosomiasis infection and prevention, and to understand the communities’ views and perspectives to facilitate the development and implementation of effective awareness strategies.

## Materials and methods

### Ethics statement

We received ethical approval from Mbarara University of Science and Technology (MUST) Research Ethical Committee (REC), reference number MUREC 1/7. We also introduced and explained the project to district authorities from whom permission to collect data was granted. Written informed consent was obtained from study participants after following an informed consent process. For both the IDIs and the FGDs, we also obtained participants’ verbal consent for audio and video recording and photo taking. Participants’ confidentiality was ensured by concealing their identities through the use of serial numbers in place of names to act as identifiers during data collection. Interviews were conducted in private and quiet places within the comfort of participants’ home settings, respecting their private matters and properties, assets and materials.

### Study design

This study adopted a fully concurrent, equal status design. The design mixes both quantitative and qualitative methods across one or more of the research processes while giving equal weight [29]. We triangulated different research methods of sampling, data collection, analysis, and discussions that enhanced completeness of the findings and helped us to explain findings from the different methods so as to answer the research question [30]. The quantitative method allowed us to assess the communities’ level of knowledge, attitude, and practices regarding schistosomiasis, while the qualitative method enabled us to investigate the reasons and motivations behind their KAPs. The use of both methods concurrently enabled us to have a wider coverage of the study population and in-depth exploration of the problem [31]. This would not have been possible with the use of one method alone.

### Study area and setting

**Fig 1:**
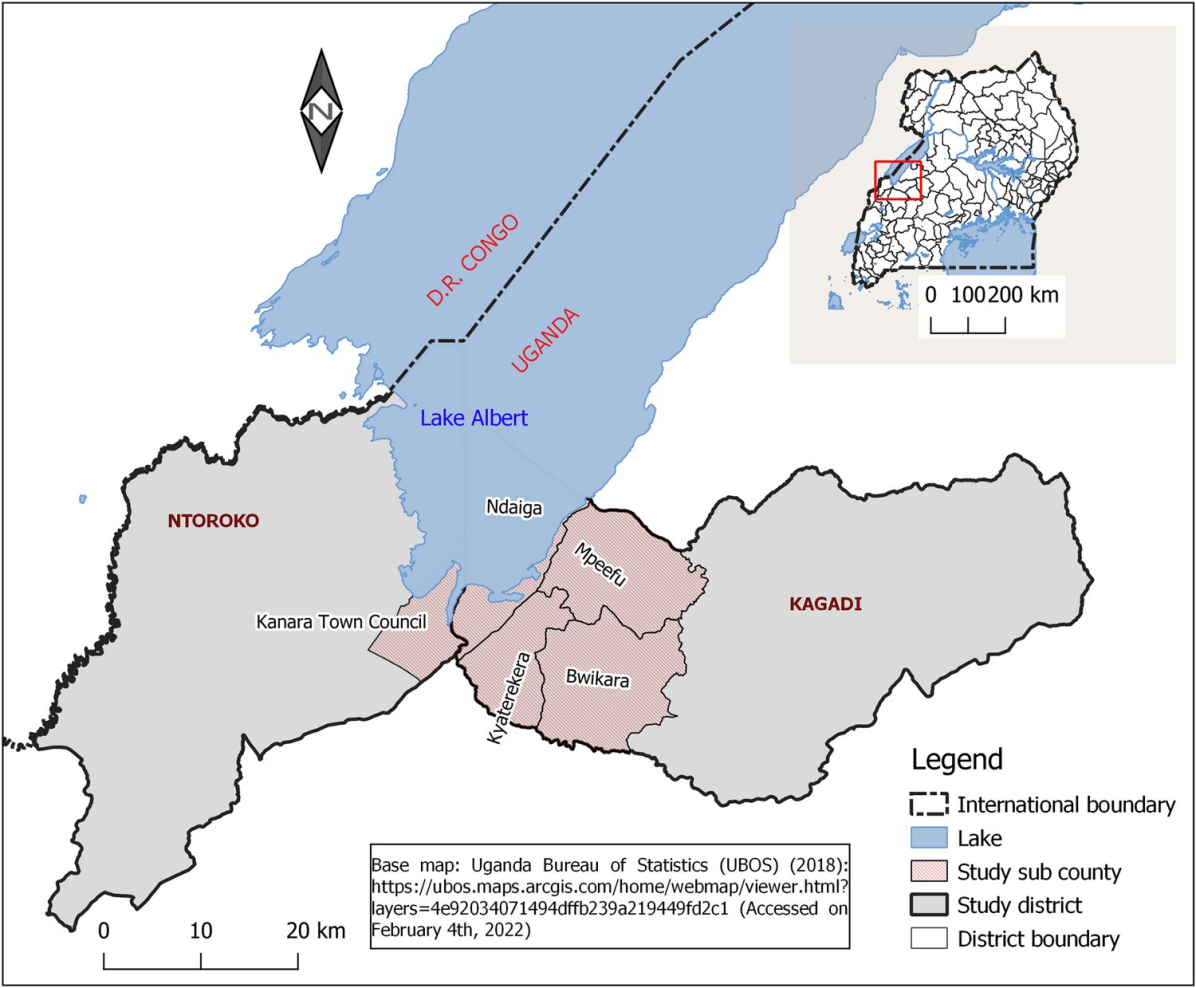
Study area showing districts and sub counties in the southern Lake Albert, Uganda. Source: Uganda Bureau of Statistics (UBOS) (2018): https://ubos.maps.arcgis.com/home/webmap/viewer.html?layers=4e92034071494dffb239a219449fd2c1 (Accessed on February 4th, 2022).

The study was conducted in Ntoroko and Kagadi districts found around Lake Albert in western Uganda between January and March 2020. We selected five sub-counties, one from Ntoroko district (Kanara Town council), and four from Kagadi district (Ndaiga, Kyaterekera, Mpeefu, and Bwikara) respectively (Fig 1). Selection was based on reported cases of schistosomiasis by the district health authority, closeness to Lake Albert, availability of other water sources such as rivers, ponds, springs, and wells, the presence of health facilities, transport networks, and human activities such as fishing, farming, and cattle rearing. Furthermore, we held meetings with the district and local leaders of the proposed study sites from where we obtained project endorsement. The area is characterized by high levels of illiteracy, gender inequality, lack of access to clean and safe water, inadequate latrine coverage, and limited health facilities [32,33]. According to our interaction with the community, citizens were supposed to receive at least one round of praziquantel a year depending on endemicity, but this had not happened in some of the villages for more than two years by the time of this study.

### Selection of participants

Study participants comprised household members who were adults of 18 years old and above, local government workers, local council chairpersons, and village health teams (VHTs). The VHTs are individuals selected and trained to conduct mobilisation and provide basic health services at the household level on a volunteer basis. For the quantitative data, we used a total sample size of 337 calculated using a formula by Kish of n = Z^2^ PQ/E^2^ [34]. Where n is the sample size, Z is the confidence level at 95% or 3 standard deviations at 1.96; P is the proportion of the population infected with schistosomiasis at 25.6% [3]; Q is the proportion of the population not infected by schistosomiasis at 74.4% (that is 1-P), and E is the standard error at 0.05 and we provided for a none response rate of 15%.

Multistage area sampling was used to select two parishes and two villages from the selected sub-counties. Disproportionate stratification was used to allocate the sample size for each village. Later, we employed a systematic sampling technique to select the households where any consenting adult member answered the questions. We first obtained a sample interval by dividing total households in a village with the sample size of each village. Thereafter, we randomly selected the first household using the fishbowl method of simple random sampling, and we selected subsequent households at intervals [35]. On the other hand, purposive sampling was used to select a total of 12 individuals for the in-depth interviews (IDIs) based on possession of adequate information about schistosomiasis, and experience in dealing with the community, while another 251 individuals comprising 28 focus group discussion (FGDs) sessions were selected in consideration of gender, residence, and accessibility.

### Data collection and analysis

Data were collected using surveys, In-depth interviews (IDIs), and Focus Group Discussions (FGDs). An interviewer-administered questionnaire was used to collect quantitative data on socio-demographic characteristics and KAP, while IDIs and FGDs were used to gather participants’ opinions and perspectives regarding the disease. For each village, we conducted two FGD sessions, one for males and another for females. Participants freely gave their opinions and perspectives, which enabled the researchers to dig deeper into the topic. With the help of the Statistical Package for Social Sciences (SPSS v.25) software, quantitative data were analysed to show frequencies, percentages, and chi-square test of associations between socio-demographic characteristics and KAPs with an alpha value of p<0.05. Thereafter, we used phi (φ), and gamma (γ) tests, which are extended chi-square measures, to determine the strength and direction of such associations. The values range from a negative one (-1) to a positive one (+1). Any phi or gamma value below 0.1 was considered to be weak, a value between 0.1 but below 0.3 as moderate, between 0.3 and 0.6 as moderately strong, and anything from 0.6 and above was considered very strong [36]. We manually analyzed qualitative data using Braun and Clerke’s seven-step model for thematic analysis [37]. Data were first transcribed, and later critically read to ensure familiarization. Manual coding was later conducted using information from both the study tools and transcripts. The codes were later developed into themes and sub-themes and grouped into different categories with those having similar meanings put together, and those having specific, unique, and different meanings put separately. A description of the themes and subthemes supported with verbatims from participants then followed. We then presented and discussed results from both quantitative and qualitative data concurrently to complement one another.

## Results

### Sociodemographic Characteristics

A total of 326 out of the 337 questionnaires giving a 96.7% response rate, were fully completed and considered for analysis. The majority of the respondents were from Ndaiga (27.3%) and Mpeefu (23.9%) sub-counties respectively. Slightly more females (52.1%) than males (47.9%) participated in the study; most of whom were aged 18–35 years (47.5%), and 36–55 years (42.9%) respectively. The majority of respondents (58.6%) were in a monogamous marriage, 55.2% had attained a primary level of education, while at least half of them had incomes less than Ug. 100,000/ = (i.e., about USD 27.17) per month **(Table 1)**.

**Table 1:** Sociodemographic characteristics of the respondents.

Description		Frequency *(N = 326)*	Percentage (%)
1	**Sub-county of residence**		
	Ndaiga	89	27.3
	Mpeefu	78	23.9
	Bwikara	61	18.7
	Kanara	61	18.7
	Kyaterekera	37	11.3
2	**Sex**		
	Female	170	52.1
	Male	156	47.9
3	**Age**		
	18–35	155	47.5
	36–55	140	42.9
	56–75	28	8.6
	76 and above	3	0.9
4	**Marital status**		
	Married (one partner)	191	58.6
	Single (not married)	60	18.4
	Married (> one partner)	44	13.5
	Separated	13	4.0
	Widowed	12	3.7
	Divorced	6	1.8
5	**Educational level**		
	Primary level	180	55.2
	Secondary level	77	23.6
	No formal education	60	18.4
	Tertiary level	6	1.8
	Post-graduate	3	0.9
**7**	**Average monthly income (UGX)**		
	Less than 100,000	164	50.3
	100,000–199,999	54	16.6
	200,000–299,000	48	14.7
	300,000–399,000	25	7.7
	400,000–499,000	10	3.1
	500,000–599,000	9	2.8
	600,000 and above	16	4.9

Key: UGX-Uganda Shillings (1$ = Ugx 3685/ =)

### Knowledge regarding schistosomiasis

Nearly all respondents (98.5%) had ever heard about schistosomiasis, mainly from family, friends, neighbors, and colleagues (28.1%). Most of the respondents (81.3%) mentioned contact with water as the main transmission mode (**Table 2)**. Participants mentioned belly enlargement (78.5%) as the common sign, while observing signs and symptoms (67.5%) as the easiest way to know whether a person has schistosomiasis. Avoiding contact with water (75.8%) was the most mentioned way to prevent schistosomiasis, followed by avoiding open defecation (51.2%) and lastly, avoiding open urination (48.8%).

**Table 2:** Respondents’ knowledge of schistosomiasis.

Description	Frequency	Percent %
**Where first heard about schistosomiasis**		
Family, friends & neighbours	89	28.1
Radio	77	24.3
Health workers	77	24.3
At the lake	21	6.6
Teachers	12	3.8
Brochures, posters, & other materials	11	3.5
Saw other people suffering	11	3.5
Have been a victim	7	2.2
Newspapers and magazines	5	1.6
TV	2	0.6
Religious leaders	2	0.6
Others	2	0.6
Billboards	1	0.3
**Signs and symptoms of schistosomiasis**		
Belly enlargement	256	78.5
Abdominal pain	194	59.5
Diarrhea	194	59.5
Blood present in the stool	149	47.5
Severe Fever	118	36.2
**Mode of schistosomiasis transmission**		
Contact with water	265	81.3
Handshakes	10	3.1
Witchcraft	3	0.9
Misfortune	2	0.6
**How to know if one has schistosomiasis**		
Signs and symptoms	220	67.5
Stool test	111	34.0
Urine test	100	30.7
Ultrasound scan	31	09.2
**Prevention methods**		
Avoid contact with water.	247	75.8
Avoid open defecation.	167	51.2
Avoid open urination.	159	48.8
Medication.	151	46.3
Avoid eating unwashed vegetables.	103	31.6

### Socio-demographic factors associated with knowledge regarding schistosomiasis

Using pearsons phi coefficient to test for the strength of association, knowledge of signs and symptoms (φ = 0.348, p = 0.006) and preventive ways (φ = 0.529, p<0.001) were found to be significantly associated with respondents’ place of residence (Table 3). Furthermore, education and knowledge of signs and symptoms (φ = 0.553, p = 0.004) had significant associations. Finally, respondents’ age and diagnosis (φ = 0.357, p = 0.024), and income and prevention (φ = 0.603, p = 0.005) were also significantly associated. **(Table 3)**.

**Table 3:** Association between respondents’ socio-demographics and knowledge of schistosomiasis.

**Description**	**φ**	**p-value**	**φ**	**p-value**	**φ**	**p-value**
	**Residence**		**Gender**		**Age**	
Signs and symptoms	.348	.006*	.113	.577	.212	.398
Transmission	.232	.398	.133	.187	.148	.601
Diagnosis	.256	.217	.093	.633	.357	.024*
Prevention	.529	.001*	.149	.723	.324	.191
	**Marital status**		**Education**		**Income**	
Signs and symptoms	.280	.432	.553	.004*	.419	.017*
Transmission	.171	.863	.230	.214	.210	.813
Diagnosis	.278	.272	.201	.580	.337	.104
Prevention	.336	.638	.298	.498	.603	.005*

Key * chi-square phi (φ) test of association was significant at p<0.05

Findings from both the quantitative and qualitative data regarding the sources of information about schistosomiasis were in agreement. Other sources of information regarding the disease mentioned by some of the FGD participants include schools, hospitals, VHTs, as well as from the local council one chairpersons, or physically seeing those with the signs and symptoms. However, some male and female FGD participants, especially those farther away from the lake, expressed ignorance of the disease, saying it was their first time to hear about it. The participants noted:

*“[Me] I had never heard about it*. *Today was my first time to hear of it*. *That’s why I kept quiet because I thought you had come to teach us about it*.” **(Female FGD, Nyakatoj, Mpeefu Sub-County)**

*“[As for me] I am a new resident in this area*. *Where I came from in Rwanda*, *I had never heard about bilharzia*. *I have spent 10 years in Uganda so far and this was my first time to hear it*.*”* **(Male FGD, Nyamarembo, Bwikara Sub-County)**

Others stated that they did not know the signs and symptoms of the disease, even though they saw people with such conditions. A male FGD participant from Songarao, Ndaiga, said:

*“I had not yet known it well*, *I am just learning now but I have seen people with the symptoms mentioned by the people above*.*”* **Male FGD Songarao, Ndaiga Sub-County**

### Local perspectives regarding schistosomiasis

Data from participants also revealed some local explanations regarding the signs and symptoms of schistosomiasis. The participants described a person with a swollen belly as a balloon or swollen part of a guitar.

*“they get swollen stomach and it becomes like a balloon; swollen cheeks; severe stomach; vomiting; swollen feet; when they are urinating they urinate urine with blood”* **Female FGD, Kayera village, Mpeefu Sub-county**

According to the participants, a person with swollen belly looks like a rich person even though they are not. Others said that due to a thin body, the person may look like someone suffering from HIV/AIDS.

*“…hands become so thin as if they have HIV/AIDs*, *cracked lips*, *and legs*.*”* **IDI-VHT Nyamasoga village-Ndaiga Sub-county**

Most people associate swollen bellies with being healthy and doing well economically, but in this context, being rich has a negative connotation. Similarly, in Uganda, some of the persons living with HIV/AIDS present with a thin body and rashes all over their skin, both of which are signs of schistosomiasis.

### Attitudes toward schistosomiasis prevention

Nearly all respondents in the sample (96.6%) agreed that schistosomiasis was a dangerous disease, 94.2% stated that it is necessary to prevent the disease. 94.8% said that they would take personal responsibility to prevent it. Almost all respondents (98.4%) agreed that defecation in a latrine was very important for their health. About three-quarters of respondents (73.4%) also acknowledged that it was important to avoid risky contact with water **(Table 4)**.

**Table 4:** Respondents’ attitudes regarding schistosomiasis.

Responses	SD	MD	DA	NS	A	MA	SA	Total
	F %	F %	F %	F %	F %	F %	F %	%
Bilharzia is a very dangerous disease.	1 (0.3%)	0 (0.0%)	2 (0.6%)	8 (2.5%)	28 (8.6%)	21 (6.5%)	265 (81.5%)	325 (100.0%)
It is necessary to prevent infection from bilharzia.	3 (0.9%)	0 (0.0%)	4 (1.2%)	12 (3.7%)	81 (24.9%)	22 6.8%	203 (62.5%)	325 (100.0%)
It is my responsibility to prevent infection from bilharzia.	3 (0.9%)	0 (0.0%)	1 (0.3%)	13 (4.0%)	95 (29.3%)	19 (5.9%)	193 (59.6%)	324 (100.0%)
It is important to avoid contact with water.	42 (13.0%)	4 (1.2%)	20 (6.2%)	20 (6.2%)	71 21.9%	11 (3.4%)	156 (48.1%)	324 (100.0%)
Defecating in the latrine is important for my health.	0 (0.0%)	0 (0.0%)	2 (0.6%)	3 (0.9%)	62 (19.1%)	17 (5.2%)	240 (74.1%)	324 (100.0%)

Key: SD-Strongly Disagree; MD-Moderately Disagree; DA-Disagree; NS-Not Sure; A-Agree; MA-Moderately Agree & SA-Strongly Agree, F-Frequency

### Socio-cultural factors associated with attitudes toward schistosomiasis

Using the gamma coefficient (γ), we measured the strength and direction of association between socio-demographic variables and attributes of attitude. We found a significantly weak negative association between the age (γ = -0.153; p = 0.046) of respondents and the perceived necessity of avoiding contact with water, but a strong negative association between income (γ = -0.308; p<0.001) of respondents, and the perceived necessity of avoiding contact with water as shown in **Table 5**. Place of residence, marital status, education, and gender did not show any significant association.

**Table 5:** Association between respondents’ sociodemographic characteristics and attitude regarding schistosomiasis.

**Description**	**γ**	**p.value**	**γ**	**p.value**	**γ**	**p.value**
	**Residence**		**Gender**		**Age**	
Schistosomiasis is a dangerous disease.	.054	.696	.054	.696	-.085	.477
It is necessary to prevent schistosomiasis.	-.054	.602	-.054	.600	-.097	.288
It is my responsibility to prevent schistosomiasis.	-.023	.824	-.023	.823	-.060	.508
It is necessary to avoid contact with water.	.077	.367	.077	.370	**-.153**	.046*
Defecation in a latrine is important for prevention.	-.025	..829	-.025	.834	.023	.831
	**Marital status**		**Education**		**Income**	
Schistosomiasis is a dangerous disease.	-.057	.620	-.069	.539	-.173	.095
It is necessary to prevent schistosomiasis.	.134	.125	.068	.179	.077	.333
It is my responsibility to prevent schistosomiasis.	.162	.060	-.010	.907	.077	.339
It is important to avoid contact with water.	.075	.317	-.029	.687	**-.308**	.001*
Defecation in a latrine is important for prevention.	.002	.983	.042	.682	.076	.117

Key * Chi-square gamma (γ*)* test of association was significant at p<0.05

#### Normalizing the abnormal of schistosomiasis

Whereas there was no significant association between perceived severity and any socio-demographics, participants from the qualitative side had mixed views, with some agreeing that schistosomiasis is a dangerous disease, while others did not. Those in favor reasoned that the disease is dangerous because it kills, has no cure, and is also stigmatizing. For example, one FGD participant said:

*“Yes*, *it is a dangerous disease because it kills*. *Once you get it*, *you can never get healed*. *It’s like having HIV*. *Most people who have suffered from it don’t live for long*. *Even people and friends run away from you once you have bilharzia*, *so madam*, *this disease is very dangerous*.*”* **Male FGD, Nyamarembo Village—Bwikara Sub County**

Meanwhile, those who felt that it is not as dangerous as other diseases are, such as Ebola or HIV/AIDS, argued that it can be prevented, it is curable, and if infected, one can live with it for a long time. They said other diseases can be treated in the hospital setting, thus indicating some knowledge of and trust in biomedical health care.

*“At least bilharzia because it is a curable disease in the area*, *but Ebola*, *AIDS are curable diseases in the area*, *but it is only when you get the treatment from the government*.*”* **IDI LCI Kitebere Village—Ndaiga Sub County**

Interestingly, both those who felt that schistosomiasis is dangerous and those who disagreed had similar reasons such as “kill” and “not kill,” “cure” and “no cure,” and liken it to having HIV/AIDS and not having. Furthermore, those who said it is dangerous still felt it is something they can live with because there is nothing they can do about it.

#### Water is our life; we cannot avoid it

Although quantitative findings revealed that avoiding contact with water is important, the qualitative side seems to suggest otherwise. For instance, some FGD participants reasoned that water is everything to them and that it is the resource from which they derive their livelihoods, including fishing and fish mongering, drinking, bathing, swimming, washing items, and many other activities. This view was held by men and women, especially from the lake communities as seen in the submissions by these FGD participants below:

*“It is very difficult to prevent ourselves from entering into the water*, *yet that is the only way through which we do survive in the area*.*”* **Male FGD, Nyamasoga Village—Ndaiga Sub County**

*“No*, *I don’t think it is possible to stop going to the water*. *All of us here are fishmongers; if you tell me to stop going to the lake*, *then I wouldn’t have what to eat the next day*. *We can try other measures*, *like not drinking the lake water and maybe defecating there*, *but we depend on the lake*.*”* **Male FGD, Ntoroko East B Village—Kanara Town Council**

In addition, whereas quantitative data revealed that defecating in a latrine is important for schistosomiasis prevention, qualitative data showed that avoiding open defecation is difficult. The male participants especially justified defecating in the lake arguing that they spend most of their time on the lake when fishing—sometimes even for a month—and therefore cannot come on land so they have to defecate in the lake. Some, however, prefer to defecate outside in an open space.

*R4*: *“Most of the men in this community are fishmongers*. *They can spend close to even a month on [the] water*. *The lake has no latrine*.*”* **Female FGD, Nyamasoga Village—Ndaiga Sub County**

### Water, sanitation, and hygiene practices (WASH) involving schistosomiasis

The majority of the respondents used lake (46.3%) or pond (23.1%) water for various purposes, including for drinking (85.3%) and washing clothes (79.1%). More than one-third (34.6%) of the respondents visit water sources either twice or ten times or more in a day, whereas 22.3% come into contact with water at least three times a day. At least 44 (13.6%) of the respondents do not have latrine facilities and thus choose to defecate in bushes, in open places, or near the water **(Table 6)**.

**Table 6:** Respondents’ practices regarding schistosomiasis.

Description	Frequency	Percent (%)
**Common sources of water (255)**		
Lake	118	46.3
Pond	59	23.1
River	34	13.3
Stream	27	10.6
A developed well	17	6.7
**Common uses of water (N = 1221)**		
Drinking	278	85.3
Washing clothes	258	79.1
Bathing	174	53.3
Washing utensils and dishes	129	39.6
Taking animals to drink	109	33.4
Fishing	98	30.1
Swimming	90	27.6
Washing vegetables	85	26.1
**Frequency of contact with water (323)**		
Thrice a day	72	22.3
Twice a day	56	17.3
10+ times/uncountable	56	17.3
Five times a day	40	12.4
Four times a day	30	9.3
Not sure	28	8.7
Once a day	24	7.4
6–9 times	15	4.6
Do not get into contact with water.	2	0.6
**Presence of latrine facility (N = 323)**		
No	44	13.6
Yes	279	86.4
**Open defecation (N = 64)**		
In the bush	40	62.5
I don’t remember.	11	17.2
In an open place	6	9.4
In the water	5	7.8
Near the water	2	3.1

### Socio-cultural factors associated with risky practices regarding schistosomiasis

Findings from the bivariate analysis, using the Pearsons phi coefficient test, revealed a significant positive association between place of residence and the commonly used sources of water (φ = 1.111; p<0.001), frequency in the use of water (φ = .578; p<0.001), and open defecation (φ = .644; p<0.001) respectively. Marital status also showed a significant positive association with commonly used sources of water (φ = .432; p = 0.012) and frequency of the use of water (φ = .451; p = 021). Finally, this study found a significant association between education and common water-related activities (φ = .505, p = 0.012) and between income and common water-related activities (φ = .439; p = 0.012) respectively. Gender and age did not show any significant association with the practices regarding WASH **(Table 7)**.

**Table 7:** Association between respondents’ socio-demographics and practices regarding schistosomiasis.

**Description**	**φ**	**p.value**	**φ**	**p.value**	**φ**	**p.value**
	**Residence**		**Gender**		**Age**	
Commonly used source of water	1.11	.001*	.098	.876	.248	.486
Common water-related activities	.267	.085	.118	.365	.160	.510
Frequency of use of water	.578	.001*	.189	.164	.196	.976
Open defecation types	.644	.001*	.337	.108	.414	.508
	**Marital status**		**Education**		**Income**	
Commonly used source of water	.432	.012*	.253	.790	.375	.308
Common water-related activities	.312	.171	.505	.012*	.439	.012*
Frequency of use of water	.451	.021*	.359	.125	.372	.577
Open defecation types	.613	.262	.511	.392	.623	.227

Key * Chi-square Phi (φ) test of association was significant at p<0.05

Data from both the quantitative and qualitative methods seemed to produce similar findings regarding risky water practices. For instance, the women especially from the lakeside complained that they do not have access to safe and clean water, which forces them to resort to the use of water from the lake. Meanwhile, those further away from the lake, said they mostly use boreholes and ponds as their sources of water. Regarding gender and usage, some FGD participants said that the men mostly use lake water for fishing purposes, whereas the women mostly use it for domestic-related purposes as seen below:

*R1*: *“This lake that you see here is the only source of water we have*.*” R2*: *“We used to have a borehole*, *but [it] got spoilt*, *so we all go to the lake*.*” R3*: *“I see men fishing*, *others buying fish from the fishermen*.*” R4*: *“Me*, *I wash from the lake*.*” R5*: *“Me*, *I bathe from the lake*, *aaarh*, *no we bath[e] at night when it’s dark and there are no people*.*” R1*: *“I have seen other people washing their clothes and utensils from the lake*.*”* **Female FGD, Nyamasoga Village—Ndaiga Sub County**

*R1*: *“Men do fish in the lake*.*” R2*: *“Women sell fish*.*” R3*: *“Women wash clothes and utensils from the lake*.*” R4*: *“Men bathe and swim in the lake*.*” R5*: *“Women fetch water to use at home*.*” R5*: *“Women collect firewood near the lake*.*” R6*: *“Women sell alcohol*.*”* **Female FGD, Songarao Village—Ndaiga Sub County**

Furthermore, although 86.4% of respondents who answered the questionnaire reported having latrines, most participants from the qualitative side especially those around the lake, explained that lack of latrines is a major problem they face. As a result, they resorted to defecating in the lake, bushes, or open spaces.

*R5*: *“But even for women*, *they defecate in the bush*, *but the feces end up in the lake*. *Even when some are bathing from the lake*, *they defecate in the lake*.*” R6*: *“Others defecate in polythene ‘kavera’ (polythene bags) and throw it behind people’s houses or compounds*. *We are doing badly*, *madam*.*”* **Female FGD, Nyamasoga Village—Ndaiga Sub County**

Other reasons for open defecation mentioned by the participants include a lack of space for construction due to a dense population, difficulties with constructing latrines due to poor soil (mostly the clay soil type is at the lakeside), and a high water table as the following participants mentioned:

*“As you can see*, *we live close to the lake*, *so if you dig a latrine*, *the water comes from deep below and break[s] it down; that’s why we have latrines far from the homes*.*”* **Male FGD, Ntoroko East B—Kanara Town Council**

*“We have some people who are just renting the place*, *and they find it difficult to construct the latrine*. *You find some other people who came from the top of the hills; they came to do fishing for two to three days and move out*, *and they do not see any reason [why] constructing the latrine is difficult*.*”* **Male FGD, Nyamasoga Village—Ndaiga Sub County**

### Myths and misconceptions regarding schistosomiasis

Findings from the qualitative data revealed several myths and misconceptions of knowledge, attitudes, and practices regarding schistosomiasis as presented below **(Table 8)**.

**Table 8:** Summary of myths and misconceptions surrounding schistosomiasis in western Uganda.

Themes	Sub-themes	Myths and misconceptions
**Knowledge**	Transmission/cause	● Being dirty causes infection. ● Eating cold and contaminated food can cause infection. ● Not washing hands when eating food can cause a person to get the disease. ● Gassing from inside of the water can cause a person to become infected.
**Practices**	Contact with water	● Have used the lake water for a long time without any health problems ● Water from deep inside of the lake is clean and safe from diseases. ● Water in the very early morning has no germs. ● Lake water tastes sweeter than water from either a borehole or water purifier (water guard).
	Open defecation	● Good for catching more fish ● Latrine defecation compromises fish caught ● Used to defecating in a bush than in a latrine

Participants noted that being dirty, gassing in the water, eating cold and contaminated food, and not washing one’s hands were some of the ways that can transmit the parasite. Similarly, some FGD participants also seem to agree as one of them said:

*“When you play in the water and gas in it*, *you can get bilarlizos (schistosomiasis)*.*”* **Female FGD, Ntoroko East B—Kanara Town Council**

A few male FGD participants, especially those along the lakeshore, also argued that they had used lake water for a long time without having any problems and therefore saw no reason to stop using it. One FGD participant had this to say:

*“For us*, *we stay next to the lake we have used water from the lake for a long time*. *We use the water for bathing*, *drinking if we go to do fishing that we have no problem if you have the money you buy if not you take the un-boiled one*. *“Polo pe dhano weng otiye malaika” (literally meaning*, *we should not expect everybody to use clean water)”* **(Male FGD, Songarao Village—Ndaiga Sub County)**

Moreover, some of the men and women explained that fetching water from the middle of the lake or very early in the morning is okay because no germs are present in that spot or during those hours of the day, respectively. A few other participants reasoned that the lake water tasted sweeter and better than water from other sources, like a borehole or water guard (purifier). One of the male participants from Songarao said:

*“But you know when you are staying in the water and you fetch the water directly in the middle of the water*. *The reason why some of us do not even boil water and you find that the feces are being carried away by the running water directly into the Lakes*.*”* **(Male FGD, Songarao Village—Ndaiga Sub County)**

Participants also said they prefer to defecate in the water because the feces act as bait for catching more fish. To them, defecating in a latrine will compromise their fish catch, and this is why they do not want to use a latrine:

*“The fishermen here believe that if you go to the latrine you don’t catch a lot of fish*. *You would rather defecate in the lake*. *Defecating in the lake helps them get a lot of fish*.*”* **Female FGD, Kitebere Village—Ndaiga Sub County**

The above myths and misconceptions were mostly held among those who stay along the shore of the lake compared with those from the hillside. Similarly, both men and women hold a similar view, although mostly the men view feces as acting as bait.

## Discussion

The continuous schistosomiasis infection and re-infections taking place in Uganda [3] could mean that some barriers to the implemented interventions still exist. We conducted a study in Kagadi and Ntoroko districts to assess the community’s knowledge, attitude, and practices, as well as to understand their views, opinions, and perspectives regarding schistosomiasis infection and prevention to help to develop and implement effective behavior change communication strategies.

### Knowledge regarding schistosomiasis

According to this study, most respondents had heard of schistosomiasis. Respondents from the lakeshore had more knowledge of signs and symptoms and preventive ways compared to those farther away. This could be attributed to the fact that the study area at the lakeside is characterized by high schistosomiasis prevalence and morbidity and therefore, that community is likely to pay more attention to the disease. This probably also explains why this study’s findings contrast with the nationwide survey that Exum and colleagues conducted, which revealed that only about two-thirds (61.8%) of respondents had heard of schistosomiasis [3]. Findings from the current study also differ from the one conducted along Lake Victoria on the Ugandan side, which showed limited knowledge of transmission routes [11]. The adequate knowledge reported in our findings could also be attributed to the recent countrywide health education campaigns by the ministry of health, Uganda between 2017 and 2018 [25].

In addition, the results from the current study show that respondents with higher income levels were more knowledgeable about the signs/symptoms and prevention compared with those with lower incomes. This is probably because, with more income, a person is likely to have more access to different sources of information regarding schistosomiasis. Findings from the study also show that educated respondents had more knowledge of the signs and symptoms than the less educated ones. This might be partly explained by the fact that people with higher education can better read communication messages that are prepared and passed in English. Furthermore, our study revealed more adequate knowledge of diagnosis of the disease among older respondents than the younger ones. It might be that with increasing age, the chances increase that people or their close relatives have experienced the disease itself, so they can tell through the signs and symptoms.

Furthermore, the current study provides an interesting insight into the community’s local perspectives regarding signs and symptoms. For instance, when a person presents with a swollen belly, people disguise it under names such as “balloon” and “guitar.” The person is also labeled as being “rich” because the swollen belly makes the person look like a rich person and yet, in a real sense, they are not. Failure to understand these local descriptions may lead to the stigmatizing and discrimination of the victims, thereby impeding prevention and control efforts. In addition, it may create a knowledge gap regarding the disease. These findings provide an interesting insight regarding the disease, which is crucial for the effective implementation of any intervention.

### Attitudes regarding schistosomiasis prevention

Findings from both the quantitative and qualitative sides revealed that generally, schistosomiasis is perceived to be a dangerous disease. The main reasons for the disease severity are long-lasting negative effects and stigma. Indeed, studies have shown that chronic schistosomiasis may induce stunting and impair cognitive development, in addition to anemia and liver complications, and many other negative effects [38]. Similarly, in other studies, like in Cameroon, Kenya, and some parts of Uganda, they found that the community perceived schistosomiasis to be a dangerous disease [13,23,39]. However, qualitative data from the current study also revealed that some people think it is not as dangerous as other diseases such as HIV/AIDS and Ebola. It is possible that those who have not had any experience of schistosomiasis or stay far away from where it is highly prevalent, tend not to bother much about it. The variations in perceptions about disease severity could also explain differences in views, opinions, and behaviors of the community regarding preventive measures. It could explain some of the myths and misconceptions regarding the disease and their behaviors as well. For example, some people who experienced the disease are normalizing it, they accept it as a fact because they see no way of avoiding it, as they depend on the water.

This study also established that although generally, the communities acknowledged that avoiding contact with contaminated water is considered to be an important preventive measure, participants especially from the lakeside, argued that it was difficult to do so. The study further revealed that the older a person is, the less likely they will think it is necessary to avoid contact with lake water and vice versa. Likewise, respondents with higher incomes were less likely to think that it is necessary to avoid contact with lake water compared to those with low incomes. This could be because the lake is where these participants obtain their living from. Furthermore, it could be that older people could have had several experiences of the disease and therefore may tend to normalize the abnormal of the disease as they feel there is nothing to do about it. But it could also be that lack of access to clean and safe water sources, coupled with low income and less education, forces these participants to rely on unclean ones such as the lake, rivers, ponds, and streams. These are some of the issues that are likely to impede any prevention effort.

Additionally, the study found that although it is important to defecate in latrines, participants said it is not possible to avoid defecating in the water because they spend most of their time on the lake. A KAP study along the Lake Victoria region of Uganda [11] and a systematic review of KAP regarding schistosomiasis in Uganda [12], also found that it was not possible to avoid contact with the water since it is linked to the community’s livelihoods. Providing clean and safe water sources and increasing latrine coverage to the community would therefore be important interventions.

### Water, sanitation, and hygiene (WASH) practices involving schistosomiasis

This study also reported the use of water from the lake and ponds for different purposes coupled with open defecation. The study found that those who reside along the lake mostly use lake water as their main source, several times a day and they practice open defecation more than those living farther away. The use of lake water was justified due to the lack of clean and safe sources of water. In addition, the failure to boil lake water was said to be due to a lack of wood fuel (scarce firewood). The continuous use of unclean water is likely to increase the risk of infection, thereby inhibiting prevention efforts. Findings from this study confirm other studies done in western Tigray-Ethiopia [15], Brazil [40], and Namibia [26], which also found that water-related activities, such as swimming, bathing, washing, and fishing, are common community practices that pose a serious risk of schistosomiasis infection.

The current study also found that those with high incomes tend to use less lake water for drinking and washing purposes compared to those that have low incomes. This could be because, with more income, they can avoid contact with the lake and use clean water from taps or boreholes, buy protective gear such as gumboots and gloves, and water purifiers and also be able to construct latrines. Equally, respondents with a higher level of education were less likely to use lake water for drinking and washing purposes than those with no or low levels of education. This could be because those with higher levels know the benefits of taking preventive measures. Indeed previous studies had also indicated that the higher one’s income the less likely they will engage in risky water practices. Likewise, those with a higher level of education tend to practice good preventive measures [40].

Furthermore, just like the findings on knowledge and attitude, this study did not find any difference in gender and risky WASH practices. That is, both male and female participants mentioned similar activities that take place at the different water sources. Qualitatively, however, participants said that the women mostly fetch water for drinking, and they also use it for cleaning fish, washing clothes and utensils, and bathing. Meanwhile, the men use it for fishing, bathing, and swimming, among other tasks. This again highlights the added value of including qualitative approaches in KAP surveys. More attention will be paid to the follow-up study to understand these conflicting results. Our findings do agree though, with another mixed-methods study conducted in Mayuge district in eastern Uganda that also showed that although quantitatively, there was no significant difference between the gender in infection, qualitatively, the adult women did most of the water contact compared to their male counterparts [18]. A deliberate effort to provide clean and safe water sources coupled with a comprehensive community sensitization that takes into consideration variations in gender, marital status, income, and education could go a long way in yielding positive outcomes.

The study also established that although quantitatively most of the respondents (86.4%) reported having adequate latrine coverage, qualitative findings seemed to suggest otherwise. It is not clear how this difference comes about, but possibly it may be that the FGD participants might have reinforced each other. But it should also be noted that having a latrine does not necessarily mean using it. The FGD participants, especially from the lakeside, also expressed that the lack of latrine facilities remains one of the biggest challenges they face. This could explain why open defecation, especially along the lake, is still a common practice. An intervention that is specifically aimed at addressing the problem of a lack of latrines, as well as problems with space and construction materials especially at the lakeside, would yield more tangible results. The findings confirm the common practices that have been reported in other studies conducted along Lake Victoria—from the Ugandan, Kenyan, and Tanzanian sides, as well as other parts of Africa [3,41–43].

### Myths and misconceptions regarding schistosomiasis

Myths and misconceptions that the community holds, such as a belief in witchcraft, shaking hands (touching the hands of a person suffering from schistosomiasis), misfortune, eating cold food, houseflies, and gassing in waters as causes of infection, were reported in this study. Additionally, the use of lake waters, especially among communities along the lakeshore, and defecating in the water to produce bait for catching more fish are some of the misconceptions reported. Previous studies in Uganda on the Lake Victoria islands also revealed some misconceptions, although they did not specify them [11]. Meanwhile, another study in the Kano state of Nigeria established that eating salty or sour foods and sharing latrines are said to be the cause of schistosomiasis [43]. As can be seen from the above, myths and misconceptions differ from place to place, and their explanations also vary. Therefore, there is a need to understand the different myths and misconceptions, as well as to design programs aimed at unpacking and debunking them in their contexts for any tangible outcomes.

### Study limitations

While the mixed method of equal weight design enabled us to assess the KAPs and to explore and understand the reasons behind such KAPs, we could not ascertain which socio-demographics, in particular, are the strongest predictors of knowledge, attitude, and practices. Future analyses should employ multivariate techniques to identify the interrelationships between the socio-demographics and test for multicollinearity. Additionally, an ethnographic study should be carried out to provide a deeper understanding of the origin and reasons behind the myths and misconceptions of the community regarding the disease.

## Conclusions and recommendations

The current study concludes that although there exists sufficient knowledge regarding schistosomiasis and positive attitudes toward its prevention, some persistent myths and misconceptions are important socio-cultural hindrances to preventive efforts. Furthermore, lack of access to clean and safe water together with low latrine coverage, are some of the factors contributing to the risky practices thereby inhibiting control and prevention of the disease. This absence of suitable alternatives is one of the reasons why the link between knowledge and practice is not always straightforward advocating for the inclusion of qualitative methods [9]. Therefore, there is a dire need for the government to provide clean and safe water sources and to ensure proper sanitation and hygiene among the communities. In addition, to address these barriers and bring down infection rates, there is a need to implement a more robust community-based awareness intervention that is culturally appropriate and context-specific to the community members’ deeply rooted habits and beliefs. Further research integrating insights from ethnographical methods, such as participant observation, could also be useful in facilitating the development of interventions for behavioral change.

## Supporting information

S1 Text Survey questionnaire. (DOC)

S2 Text In-depth interview guide. (DOC)

S3 Text Focus group discussion guide. (DOC)

S4 Text Informed consent form. (DOC)
