# Supplementary material for: Knowledge, attitudes, and practices regarding schistosomiasis infection and prevention: A mixed-methods study among endemic communities of western Uganda
Source: PLoS Negl Trop Dis. 2022 Feb 23;16(2):e0010190. doi: 10.1371/journal.pntd.0010190 (PMC8865686; doi:10.1371/journal.pntd.0010190)
Supplement: S1 Text — (DOC) [file pntd.0010190.s001.doc]

**Community Engagement and Schistosomiasis Prevention: A Sociological Analysis of the Citizen Science Model among Selected Communities of Western Uganda**

**Respondent Code: ……………………**

**Name of village…………………… Parish…………………….. Sub-county……………….**

**INSTRUCTION: Please read the questions carefully and tick or circle the letter/s that correspond/s to the correct answer**

1. **SOCIO-DEMOGRAPHIC CHARACTERISTICS**
2. **What is your gender?**
3. Male
4. Female
5. **How old are you?..........................** *(age in years only)*
6. **What is your marital status?**
7. Single (not married)
8. Married (spouse has one partner)
9. Married (spouse has more than one partner)
10. Widowed
11. Divorced
12. Separated
13. Others specify…………
14. **If you are married, what form of marriage?**
    1. Customary
    2. Civil
    3. Religious
15. **What is your highest level of education?**
16. No formal education
17. Primary level
18. Secondary level
19. Tertiary level
20. Post-graduate
21. **Which of this is your ethnicity?**
    1. Mutoro/Mutuku
    2. Mukiga
    3. Munyoro
    4. Mukonjo
    5. Mufumbira/Munyarwanda
    6. Alur
    7. Mwamba
    8. Other, specify………....
22. **Which kind of affiliation is customary in your clan?**
    1. Matrilineal (mother’s side)
    2. Patrilineal (father’s side)
    3. Bilineal
23. **What is your religious affiliation?**
    1. Catholic
    2. Anglican
    3. Muslim
    4. Pentecostal/Born Again/Evangelical
    5. Seventh Day Adventist
    6. Traditionalist
    7. Faith of unity
    8. Orthodox
    9. Others specify……………..
    10. Non-Religious
24. **What is your main source of income?**
    1. Crop growing
    2. Fishing
    3. Fish farming
    4. Livestock rearing/animal husbandry
    5. Business
    6. Employment
    7. Others specify……………………
25. **What is your average monthly income?**
    1. Less than Ugx: 100,000/=
    2. Ugx: 100,000/=-Ugx: 199,999/=
    3. Ugx: 200,000/=- Ugx: 299,000/=
    4. Ugx: 300,000/=-Ugx: 399,000/=
    5. Ugx: 400,000/= Ugx: 499,000/=
    6. Ugx: 500,000/=- Ugx: 599,000/=
    7. Ugx: 600,000/= and above
26. **How many members are in your household?..........................**
27. **What is the estimated distance from the nearest health facility?** *(in kilometres) ......*
28. **How many water sources (domestic and others) are present in the village?............**
29. **What is the estimated walking time to reach the nearest source of water for domestic use by your household? (estimate in minutes) ...................................**
30. **PRACTICES TOWARDS BILHARZIA**
31. **Which of these is the most commonly used source of water?** *(Tick all that apply)*
32. River
33. Lake
34. Stream
35. Pond
36. A developed well
37. An undeveloped well
38. Borehole
39. Piped tape
40. Other……………….
41. **Which kind of activities are carried out at the respective water sources selected above?** *(tick all that apply)*
    1. Drinking water
    2. Washing clothes
    3. Washing utensils and dishes
    4. Washing vegetables
    5. Fishing
    6. Bathing
    7. Taking animals for drinking
    8. Swimming
    9. Washing motorcycles
    10. Defecation
    11. Urination
    12. Playing by children
    13. Irrigation
    14. Others specify………….
42. **On average, how many times in a day do you get into contact with the water?**
    1. Once a day
    2. Twice a day
    3. Thrice a day
    4. Four times a day
    5. Five times a day
    6. Others specify.....
    7. Do not get into contact with water
    8. Not sure
43. **Do you have a toilet facility? (If no, go to question #23)**
44. No (Give reason(s)………………………………………………)
45. Yes
46. **When was the last time you defecated outside the toilet?**
    1. Less than a week a go
    2. More than a week ago but within a month
    3. More than a month ago but within the last three months
    4. More than three months ago but within the last six months
    5. More than six months ago but within this year
    6. More than a year ago
    7. I can’t remember
    8. I have never defecated outside the toilet
47. **Where exactly did you defecate outside the toilet?**
    1. In an open space
    2. In the bush
    3. In the water
    4. Near the water
    5. I don’t remember
48. **How often do you defecate outside the toilet?**
    1. Quite often
    2. Less often
    3. Rarely
    4. It was only once
    5. As and when nature calls
    6. Don’t know
    7. Other specify………..
49. **What would be the main reason for you defecate outside the toilet?**
    1. I don’t have toilet
    2. I don’t like using toilet
    3. The toilet is very far
    4. The toilet is spoilt
    5. My culture doesn’t allow use of toilet
    6. Because of need to respond to nature’s call
    7. Other specify……….
50. **When was the last time you urinated outside the toilet?**
    1. Less than a week a go
    2. More than a week ago but within a month
    3. More than a month ago but within the last three months
    4. More than three months ago but within the last six months
    5. More than six months ago but within this year
    6. More than a year ago
    7. I can’t remember
    8. I have never urinated outside the toilet
51. **Where exactly did you urinate outside the toilet?**
    1. In an open space
    2. In the water
    3. In the bush
    4. I don’t remember
52. **How often do you urinate outside the toilet?**
    1. Quite often
    2. Less often
    3. Rarely
    4. It was only once
    5. As and when nature calls
    6. Other specify…………..
53. **What would be main reason for you to urinate outside the toilet?**
    1. I don’t have toilet
    2. I don’t like using toilet
    3. The toilet is spoilt
    4. My culture doesn’t allow use of toilet
    5. Because I am pressed by nature’s call
    6. Other specify……….
54. **KNOWLEDGE AND AWARENESS OF BILHARZIA**
55. **Have you ever heard about bilharzia?** *(If no go to question 34)*
56. No
57. Yes
58. **If you have ever heard of bilharzia, where did you first hear about it** *(Tick only one option)*
    1. Newspapers and magazines
    2. Radio
    3. TV
    4. Billboards
    5. Brochures, posters and other printed materials
    6. Health workers
    7. Family, friends, neighbours and colleagues
    8. Religious leaders
    9. Political leaders
    10. Teachers
    11. Other ………………….
    12. Never heard of bilharzia
59. **If you have heard about bilharzia, which of the following is the correct meaning of bilharzia?** *(Tick only one response)*
60. It is a disease
61. It is a curse
62. It is a symptom
63. Others specify……………………….
64. None of the above
65. Don’t know
66. **If you have heard about bilharzia, what are the signs and symptoms of bilharzia?** *(Please Tick all that apply)*
67. Abdominal pain
68. Diarrhoea
69. Blood present in stool
70. Belly enlargement
71. Severe Fever
72. Skin rash
73. Stunted growth
74. Reduced learning ability
75. Other specify…………………
76. Do not know
77. **If you have heard about bilharzia, how can a person get bilharzia?** *(Please Tick all that apply)*
    1. Handshakes
    2. Contact with water
    3. Witchcraft
    4. Misfortune
    5. Violating traditional taboos
    6. Do not know
    7. Other…………………..
78. **How can a person know that they have bilharzia?** *(Tick all that apply)*
    1. Signs and symptoms
    2. Urine test
    3. Stool test
    4. Ultrasound scan
    5. Other specify……………
    6. I don’t know
79. **How can a person prevent infections from bilharzia?** *(Please Tick all that apply)*
    1. Medication
    2. Avoiding contact with water
    3. Avoid open defecation
    4. Avoid open urination
    5. Avoid eating unwashed vegetables
    6. Avoid uncooked meat
    7. Snail control
    8. Do not walk barefoot
    9. Boiling water from river or lake
    10. Other specify…..………
80. **Have you or any of your family members had any of the following symptoms?** *(Read all responses)*
    1. Abdominal pain
    2. Diarrhoea
    3. Blood present in stool
    4. Belly enlargement
    5. Severe Fever
    6. Skin rash
    7. Stunted growth
    8. Reduced learning ability
    9. Other specify…………………
    10. Do not know
81. **ATTITUDES TOWARDS SCHISTOSOMIASIS**

***Now I would like you to give your opinion on schistosomiasis by stating the extent to which you agree or disagree with each of the following statements. The options range from Strongly Agree (SA), Moderately Agree (MA), Agree (A), Not Sure (NS), Dis-Agree (DA), Moderately Disagree, Strongly Disagree (SD)***

| **Qn** | **Statement** | **SA** | **MA** | **A** | **NS** | **DA** | **MD** | **SD** |
| --- | --- | --- | --- | --- | --- | --- | --- | --- |
|  | Bilharzia is a very serious disease |  |  |  |  |  |  |  |
|  | It is necessary to prevent infection from bilharzia |  |  |  |  |  |  |  |
|  | It is my responsibility to prevent infection from bilharzia |  |  |  |  |  |  |  |
|  | It is important to know whether I have bilharzia or not |  |  |  |  |  |  |  |
|  | It is important to avoid contact with water |  |  |  |  |  |  |  |
|  | Defecating in the toilet is important for my health |  |  |  |  |  |  |  |
|  | Urinating in the toilet is important for my health |  |  |  |  |  |  |  |
|  | I sometimes eat uncooked meat |  |  |  |  |  |  |  |
|  | I eat unwashed vegetables quite often |  |  |  |  |  |  |  |
|  | I would take action if I found that I had bilharzia |  |  |  |  |  |  |  |
|  | Taking medication for bilharzia is important for my health |  |  |  |  |  |  |  |
|  | If I find that have bilharzia, I should go to hospital |  |  |  |  |  |  |  |
|  | If I find that have bilharzia, I should see a traditional health practitioner |  |  |  |  |  |  |  |
|  | It is important for me to be informed about bilharzia |  |  |  |  |  |  |  |
|  | I would wish to get more information about bilharzia |  |  |  |  |  |  |  |

1. **SCHISTOSOMIASIS INFECTIONS, RE-INFECTIONS AND HEALTH-SEEKING BEHAVIOUR**
2. **In the past one year, have you ever suffered from bilharzia?** *(if your response is no, skip to Q52)*
   1. No
   2. Yes
3. **How many times have you ever suffered from bilharzia?**.................
4. **If no, has any of your family members ever experienced any of the following symptoms?**
   1. Abdominal pain
   2. Diarrhoea
   3. Blood present in stool
   4. Belly enlargement
   5. Severe Fever
   6. Skin rash
   7. Stunted growth
   8. Reduced learning ability
   9. Other specify…………………
   10. Do not know
5. **How many of the members have ever experienced the above symptoms?......................**
6. **In the past one year, did you or any of the relatives seek help from anywhere about the symptoms?**
   1. No (**If no, why?..............................................................................................)**
   2. Yes
7. **If yes, where did you seek help from?** *(Tick all that apply)*
8. Health facility
9. Traditional health practitioner
10. Treated my self
11. Others specify………………………………
12. **If you sought help from health facility, which facility was it?** *(Tick all that apply)*
    1. Private for-profit clinic
    2. Private not for profit health facility
    3. Government health facility (like HCII, HCIII or HCIV)
    4. Bought medicines from drug shop/pharmacy (mention drug…………………………..)
    5. Other specify…………………..
13. **If you sought help from traditional health practitioner, which of the following did you seek help from?** *(Tick only one option)*
    1. Witch doctor
    2. Traditional birth attendant
    3. Took herbal medicine (specify……………………………..)
    4. Other specify………………..
14. **If you treated yourself, what exactly did you do?**
    1. I bought some drugs
    2. I got some herbs
    3. I prayed

**F. SCHISTOSOMIASIS INFORMATION & COMMUNICATION**

1. **Which of the following information about bilharzia would you wish to get most?** (Mention all)
2. Causes
3. Signs and symptoms
4. Prevention
5. Cure/Treatment
6. Care for the sick
7. Drugs availability, accessibility & affordability
8. Side effects of drugs
9. Myths and misconceptions
10. Where to report cases/symptoms
11. Others ........................................
12. Don’t know
13. **Which of the following sources of information on bilharzia do you think can most effectively reach you?** (*Please choose only three most effective sources*)
    1. Newspapers and magazines
    2. Radio stations
    3. TV channels
    4. Theatre plays
    5. Mobile Phone messages
    6. Billboards
    7. Brochures, posters and other printed materials
    8. Health workers
    9. Family, friends, neighbours and colleagues
    10. Religious leaders
    11. Politicians
    12. Famous actors or singers
    13. Teachers
    14. Other (specify)…………………………………………………………….
14. **Do you have anything else to say?**
15. No
16. Yes

If yes state here……………………………………….………………………………………

***Thank you very much for your participation in the study!!***
