# Supplementary material for: Knowledge, attitudes, and practices regarding schistosomiasis infection and prevention: A mixed-methods study among endemic communities of western Uganda
Source: PLoS Negl Trop Dis. 2022 Feb 23;16(2):e0010190. doi: 10.1371/journal.pntd.0010190 (PMC8865686; doi:10.1371/journal.pntd.0010190)
Supplement: S2 Text — (DOC) [file pntd.0010190.s002.doc]

***Topic: Community engagement and schistosomiasis prevention: A sociological analysis of the citizen science approach among selected rural communities of western Uganda***

**IN-DEPTH INTERVIEW GUIDE FOR WITH SELECTED COMMUNITY MEMBERS & LEADERS**

1. Introduction of interviewer/researcher and purpose of the interview
2. **Demographic Information-**Briefly introduce yourself in terms of your age, gender, level of education, working status, marital status and nearest health facility from your home (probe for any information not mentioned).
3. **Practices regarding schistosomiasis**

- Explain for me the common sources of water in your area and their different uses? (probe for each source of water mentioned)
- What type of latrines do you have in your homestead? Explain their conditions, and how you use them *(probe for type, defecation and urination, use by age, gender, etc.)*

1. **Knowledge-In your own understanding, what do you know about** **bilharzia**?
   - Briefly tell me what you know about bilharzia i.e. what it is, signs & symptoms, risk factors etc
   - What are some of the water related activities that lead to infection from bilharzia and how?
   - How does defecating outside the toilet lead to infection from bilharzia?
   - How does urinating outside the toilet cause infection from bilharzia?
   - What should a person infected with bilharzia do?
   - What medications are given to people who are infected with bilharzia (probe for type, availability, affordability, accessibility, etc.)
   - In your opinion how can a person prevent infection from bilharzia?
   - In what ways can the community get involved in prevention of infection from bilharzia?
   - What information about bilharzia would you need to be given? And how would you need to receive information about bilharzia?
2. **Attitudes**

- In your opinion do you think bilharzia is a serious disease? If so, why? And if not, why?
- Personally, do you think it is necessary for you to be bothered about bilharzia? Why?
- Some people say that it is necessary to prevent bilharzia infection? What do you have to say about it?
- What would you do if you found that either you or your close relative had bilharzia?
- When was the last time you checked for bilharzia? If not, would you be willing to check for bilharzia?
- In your opinion how important is it to defecate or urinate in toilet?
- What is your opinion about avoiding contact with water?

1. **Health-seeking behaviour**

- What actions do you take when you fall sick or feel unwell? *(probe for place of medication-hospital, private clinic, pharmacies/drug shops, traditional medication, prayers, finance, distance, gender dynamics etc.)?*
- What actions did you or your close relative take when you or that person found that you or the person had bilharzia?
- Where do people in your community go when they find that they have bilharzia or its symptoms?
- What reasons do people give for taking medications for bilharzia from where they go

1. **Awareness and behaviour change campaign strategy**
   - Where do you currently get health information from, which of the sources of health information do you trust most and why?
   - If you have ever heard about bilharzia, where did you first hear from?
   - If you have ever heard of bilharzia or if not, we would like to make sure more people receive correct information about bilharzia, in your opinion in which way can we best spread information on bilharzia in order to reach as many people as possible?
2. **Gender dynamics and complexities**

- What are some of the household assets and who has ownership over them and why?
- Which activities are mostly done by women (and girls) and which ones are done by men (and boys)?
- Who decides on what should be done in the household and why?
- Which water related activities are specifically done by women and which ones are done by men? And who decides on the water related activities done by each of the gender and why?
- Between women and men, who is more likely to get bilharzia and why?

1. **Conclusion:** Thank you very much for taking your time to participate in this study; before we end the conversation, I would like to know whether you have any feed back to the conversation and whether you have anything else to say.

***Thank you very much the conversation has ended.***
