# Supplementary material for: Knowledge, attitudes, and practices regarding schistosomiasis infection and prevention: A mixed-methods study among endemic communities of western Uganda
Source: PLoS Negl Trop Dis. 2022 Feb 23;16(2):e0010190. doi: 10.1371/journal.pntd.0010190 (PMC8865686; doi:10.1371/journal.pntd.0010190)
Supplement: S3 Text — (DOC) [file pntd.0010190.s003.doc]

***Topic: Community engagement and schistosomiasis prevention: A sociological analysis of the citizen science approach among selected rural communities of western Uganda***

**FGD GUIDE WITH SELECTED COMMUNITY MEMBERS FOR KAPs STUDY**

1. **Introduction:** The research team, purpose of the meeting including some brief background to the study
2. **Demographic information**

- I would like to ask you to start by giving me your background in terms of age, gender, level of education, working status, and marital status and nearest health facility from your home (probe for any information not mentioned).

1. **Practice**

- Which sources of water do you have and what common activities are carried out at each of the different water sources? (probe for fishing, bathing, swimming, washing utensils, clothes etc.)
- Do people in your community have latrines? Which type and how do you use them? what challenges do you face with them
- Are there some people in your community that defecate or urinate in the open? (probe for bush, water, road etc) If so, which age group and gender is the most common and why? Why would they defecate or urinate in the bush?

1. **Knowledge of bilharzia**

- In your own understanding, tell me what you know about bilharzia (signs and symptoms)
- How do people get infected with bilharzia and which categories of people can get infected?
- Do you think that a person who has ever been infected earlier can get it again or not? If so, why and how?
- Explain ways in which bilharzia can be prevented
- What are the available treatments, where and the associated treatment costs?
- Describe some of the water related health problems of this community (probe if bilharzia is not mention)
- How does defecating and or urinating in open/water related to bilharzia?

1. **Community attitudes towards bilharzia**

- Some people say bilharzia is a very serious disease, what do you have to say about it and why?
- How would you react to signs and symptoms of bilharzia and Why?
- In your own capacity what would you do to prevent infection from bilharzia?
- What is your opinion on the idea of avoiding contact with water in order to prevent infection from bilharzia and Why? (probe for swimming, fetching water, washing clothes and utensils, etc)
- Do you think defecating or urinating outside toilet is bad? If so why? Why do some people still defecate and or urinate outside the toilet?
- What actions would you take if you or a close member of your household had bilharzia?
- In your opinion, would it be necessary to get any information about bilharzia? If so, why?
- When was the last time you checked for bilharzia? If not, would you be willing to check for bilharzia?

1. **Health-seeking behaviour**

- Symptoms experience-how does the community know when a person has bilharzia?
- What do you do when you discover you have bilharzia? Who decides on what to do?
- Health seeking on bilharzia –what medications do you take for bilharzia?
- Where do you go for medication for bilharzia?

1. **Gender and children**

- Risk of exposure- how do gender differences in water related activities influence risk of bilharzia infection?
- Gender dimensions of health seeking-decision making; Which medications do the different genders take and where do they go to for medication?
- What support would the men give if their wives or children got infected with bilharzia? (probe for escorting women, taking children to hospital, financial support, social support, psychological support etc.)

1. **Information and communication**

- What are the most commonly used sources of health information by the community currently?
- Which sources of health information would you prefer and why?
- When was the first time you heard information about bilharzia? What is your view current information about bilharzia in terms of ease of accessibility, affordability, availability etc?
- In your opinion, what is the best way of spreading information on bilharzia in your community?

1. **Conclusion:** Thank you very much for taking your time to come and participate in this important discussion on how we can address the problem of bilharzia. Before we end, I would like to know if anyone of you has any further contribution to make.

***Thank you very much the meeting has ended.***
