# Supplementary material for: Knowledge, attitudes, and practices regarding schistosomiasis infection and prevention: A mixed-methods study among endemic communities of western Uganda
Source: PLoS Negl Trop Dis. 2022 Feb 23;16(2):e0010190. doi: 10.1371/journal.pntd.0010190 (PMC8865686; doi:10.1371/journal.pntd.0010190)
Supplement: S4 Text — (DOC) [file pntd.0010190.s004.doc]

## Appendix I: Informed Consent Form for Household Questionnaire to respondents

**MBARARA UNIVERSITY OF SCIENCE AND TECHNOLOGY**

**RESEARCH ETHICS COMMITTEE**

P.O. Box 1410 Mbarara, Tel: +256-48-543-3795, Fax: +256-48-542-0782

E-mail : [irc@must.ac.ug](mailto:irc@must.ac.ug), [mustirb@gmail.com](mailto:mustirb@gmail.com)

| **Study title:** Community Engagement and Schistosomiasis Prevention: A Sociological Analysis of the Citizen Science Model among Selected Communities of Western Uganda |
| --- |

Principal Investigator(s)

| Anyolitho Maxson Kenneth |
| --- |

**Introduction**

Hello, I am a student of Mbarara University of Science and Technology (MUST) pursuing a PhD

study in development studies. It is part of a five-year project (January 2019 to December, 2023)

named: Action Towards Reducing Aquatic snail born Parasitic diseases (ATRAP) with the main

goal of this project is to aid in the prevention of snail-borne diseases in Uganda thereby lowering

the socio-economic burden created by these diseases. The project that is being implanted in the

two districts of Kagadi and Ntoroko in Western Uganda, is a collaboration between Mbarara

University of Science and Technology (MUST) and the Belgium government.

Brief background to the study

| The interview, for which you are being asked to participate in, is a study that focuses on the prevention of schistosomiasis disease in Uganda. It is hoped that the outcome of the study will greatly help in the prevention of schistosomiasis by bringing positive change in behaviors of the community. |
| --- |

**Purpose of the research project**

The purpose of this study is to examine the role of citizen science model in community

engagement to the prevention schistosomiasis among selected rural communities of Western Uganda. The study objectives are: to determine community knowledge, attitude and practices regarding schistosomiasis, their health seeking behaviors, practices and attitudes, lived experiences and perceptions regarding schistosomiasis, implementation of contextualized. The methods that will be used to meet this purpose include: Administering a survey questionnaire to 337 adult household members and to another 337 adult household members randomly selected from the study area for the baseline study and for the end line study respectively. The estimated duration for administering the questionnaire shall take between 45 minutes and one hour.

**Why you are being asked to participate?**

You have been asked to participate in the study on the basis that you know and understand the

importance of participating in the study and that you will be able to provide the necessary

information required for the study.

**Procedures**

The data collection procedure shall involve you answering questions that shall be put forward to you by the enumerator in a question-and-answer session on your background characteristics, and your opinions about knowledge, attitude, and practices including health seeking behaviours, lived experiences and perceptions regarding schistosomiasis.

**Risks or discomforts**

There are no any known or anticipated risks associated with your participation in the study. Where such risks are known or shall be discovered in the process of the study, they shall be kept to bare minimum possible.

**Benefits**

There are no any specific benefits of this study but generally, findings of the study will help: To

improve community knowledge and attitude towards schistosomiasis; to identify and address

social and cultural factors that predispose schistosomiasis infection, to create awareness on

schistosomiasis, and to scale up community involvement in schistosomiasis prevention.

**Incentives or rewards for participating**

There are no any incentives or rewards attached to this study.

**Protecting data confidentiality**

Any information shared with the research team will be treated as confidential. When processing

and publishing data, anonymity is guaranteed, as all data will be coded. This means that data that

can identify a respondent will be systematically ‘translated’ into coded that will not identify the

person who shared the data.

**Protecting subject privacy during data collection**

All Interview sessions shall be conducted in a private and quiet place chosen by the

respondent/participant. Any sensitive question that may negative affect the respondent shall not

be asked.

**Right to refuse or withdraw**

Your participation in this study is voluntary, you have the right not to participate in the study if

you so wish or to leave at any moment. There is no any penalty or loss of benefits to which you

would be entitled even if you decide not to participate.

**What happens if you leave the study?**

There is no penalty that shall be levied to you if you decide to leave this study at any moment of

the session. You may withdraw from this study at any time. There is no obligation to participate

in this project.

**Compensation and Refreshments**

As part of expenses regarding the data collection, each participant will be given Ugx 5,000=

(Five thousand only) as compensation for the time spent while participating in the study.

Additionally, a participant will where appropriate, be given Ugx 10,000= (Ten Thousand

Shillings only) as reimbursement for transport expenses.

**Who do I ask/call if I have questions or a problem?**

In case you have any questions related to the study, you may contact the chairperson of Mbarara

University of Science and Technology (MUST) Research and Ethics Committee (REC) Dr.

Francis Bajunirwe on Tel. no. 0772576396/ 0485433795. For any questions related to your rights

and privacy, you may contact me on Tel 0776630123, or my Supervisor Dr. Viola Nilah Nyakato

on Tel. 0772982535

**What does your signature or thumbprint on this consent form mean?**

Your signature on this form means that you have read and understood the informed consent

information which includes but is not limited to the study’s purpose, procedures, possible

benefits and risks and that you have been given the chance to ask questions before you sign. You

have also had the opportunity to ask questions about the study and any questions that you have

asked have been answered to your satisfaction and that you voluntarily consent to be a

participant in the research.

_ _ _ _ _ _ _ _ _ _ _ _ _ _ _ _ _ _ _ _ _ _ _ _ _ _ _ _ _ _ _ _ _ _ _ _ _ _ _ _ _

Name of adult participant Signature of participant or Date

Legally authorized representative

_ _ _ _ _ _ _ _ _ _ _ _ _ _ _ _ _ _ _ _ _ _ _ _ _ _ _ _ _ _ _ _ _ _ _ _ _ _ _

Name of person obtaining consent Signature Date

_ _ _ _ _ _ _ _ _ _ _ _ _ _ _ _ _ _ _ _ _ _ _ _ _ _ _ _ _ _ _ _ _ _ _ _ _ _

Print Name of witness Signature or thumbprint or mark Date

## Appendix Three: Informed Consent Form for Focus Group Discussions (FGDs)

**MBARARA UNIVERSITY OF SCIENCE AND TECHNOLOGY**

**RESEARCH ETHICS COMMITTEE**

P.O. Box 1410 Mbarara, Tel: +256-48-543-3795, Fax: +256-48-542-0782

E-mail : [irc@must.ac.ug](mailto:irc@must.ac.ug), [mustirb@gmail.com](mailto:mustirb@gmail.com)

| **Study title:** Community Engagement and Schistosomiasis Prevention: A Sociological Analysis of the Citizen Science Model among Selected Communities of Western Uganda |
| --- |

Principal Investigator(s)

| Anyolitho Maxson Kenneth |
| --- |

**Introduction**

Hello, I am a student of Mbarara University of Science and Technology (MUST) pursuing a PhD

study in development studies. It is part of a five-year project (January 2019 to December, 2023)

named: Action Towards Reducing Aquatic snail born Parasitic diseases (ATRAP) with the main

goal of this project is to aid in the prevention of snail-borne diseases in Uganda thereby lowering

the socio-economic burden created by these diseases. The project that is being implanted in the

two districts of Kagadi and Ntoroko in Western Uganda, is a collaboration between Mbarara

University of Science and Technology (MUST) and the Belgium government.

Brief background to the study

| The Focus Group Discussions, for which you are being asked to participate in, is part of a study that focuses on the prevention of schistosomiasis disease in Uganda. It is hoped that the outcome of the study will greatly help in the prevention of schistosomiasis by bringing positive change in behaviors of the community. |
| --- |

**Purpose of the research project**

The purpose of this study is to examine the effectiveness of citizen science model in community

engagement to the prevention of schistosomiasis among selected rural communities of Western

Uganda. The study objectives are: to determine community knowledge, attitude and practices

regarding schistosomiasis, their health seeking behaviors, practices and attitudes, lived

experiences and perceptions regarding schistosomiasis, implementation of contextualized. The

methods that will be used to meet this purpose include: conducting Focus Group Discussions

(FGDs) with selected members of the community of the study area. The estimated duration for

each of the sessions shall take between One hour and One hour thirty minutes.

**Why you are being asked to participate?**

You have been asked to participate in the study on the basis that you know and understand the

importance of participating in the study and that you will provide the necessary information

required for the study.

**Procedures**

The data collection procedure shall involve your participation by giving views, opinions and thoughts about the study in a group discussions format that is expected to be interactive in nature. You are also informed that there is no right or wrong answer. We value every response that will be provided. Discussions shall focus on topics of knowledge, attitude, and practices including health seeking behaviours, lived experiences and perceptions regarding schistosomiasis. Each FGD comprises between eight and ten participants selected from the community on the categorised into sex. For proper collection of data, all sessions will be recorded both in a note book and or in an audio-recording device.

**Risks or discomforts**

There are no any known or anticipated risks associated with your participation in the study. Where such risks are known or shall be discovered in the process of the study, they shall be kept to bare minimum possible.

**Benefits**

There are no any specific benefits of this study but generally, findings of the study will help: To

improve community knowledge and attitude towards schistosomiasis; to identify and address

social and cultural factors that predispose schistosomiasis infection, to create awareness on

schistosomiasis, and to scale up community involvement in schistosomiasis prevention.

**Incentives or rewards for participating**

There are no any incentives or rewards attached to this study.

**Protecting data confidentiality**

Any information shared with the research team will be treated as confidential. When processing

and publishing data, anonymity is guaranteed, as all data will be coded. This means that data that

can identify a respondent will be systematically ‘translated’ into coded that will not identify the

person who shared the data.

**Protecting subject privacy during data collection**

All FGD sessions shall be conducted in a private and quiet place chosen by the

respondent/participant. Any sensitive question that may negative affect the respondent shall not

be asked.

**Voluntarism**

Your participation in this study is voluntary, you have the right not to participate in the study if

you so wish or to leave at any moment. There is no any penalty or loss of benefits to which you

would be entitled even if you decide not to participate.

**What happens if you leave the study?**

There is no penalty that shall be levied to you if you decide to leave this study at any moment of

the session. You may withdraw from this study at any time. There is no obligation to participate

in this project.

**Compensation and Refreshments**

As part of expenses regarding the data collection, each participant will be given Ugx 5,000=

(Five thousand only) as compensation for the time spent while participating in the study.

Additionally, a participant will where appropriate, be given Ugx 10,000= (Ten Thousand

Shillings only) as reimbursement for transport expenses. Participants will also be provided with

refreshment in terms of either sodas or water during the FGD sessions.

**Who do I ask/call if I have questions or a problem?**

In case you have any questions related to the study, you may contact the chairperson of Mbarara

University of Science and Technology (MUST) Research and Ethics Committee (REC) Dr.

Francis Bajunirwe on Tel. no. 0772576396/ 0485433795. For any questions related to your rights

and privacy, you may contact me on Tel 0776630123, or my Supervisor Dr. Viola Nilah Nyakato

on Tel. 0772982535

**What does your signature or thumbprint on this consent form mean?**

Your signature on this form means that you have read and understood the informed consent

information which includes but is not limited to the study’s purpose, procedures, possible

benefits and risks and that you have been given the chance to ask questions before you sign. You

have also had the opportunity to ask questions about the study and any questions that you have

asked have been answered to your satisfaction and that you voluntarily consent to be a

participant in the research.

_ _ _ _ _ _ _ _ _ _ _ _ _ _ _ _ _ _ _ _ _ _ _ _ _ _ _ _ _ _ _ _ _ _ _ _ _ _ _ _ _

Name of adult participant Signature of participant or Date

Legally authorized representative

_ _ _ _ _ _ _ _ _ _ _ _ _ _ _ _ _ _ _ _ _ _ _ _ _ _ _ _ _ _ _ _ _ _ _ _ _ _ _

Name of person obtaining consent Signature Date

_ _ _ _ _ _ _ _ _ _ _ _ _ _ _ _ _ _ _ _ _ _ _ _ _ _ _ _ _ _ _ _ _ _ _ _ _ _

Print Name of witness Signature or thumbprint or mark Date

**MBARARA UNIVERSITY OF SCIENCE AND TECHNOLOGY**

**RESEARCH ETHICS COMMITTEE**

P.O. Box 1410 Mbarara, Tel: +256-48-543-3795, Fax: +256-48-542-0782

E-mail : [irc@must.ac.ug](mailto:irc@must.ac.ug), [mustirb@gmail.com](mailto:mustirb@gmail.com)

## INFORMED CONSENT FORM FOR IN-DEPTH INTERVIEWS (IDIs)

| **Study title:** Community Engagement and Schistosomiasis Prevention: A Sociological Analysis of the Citizen Science Model among Selected Communities of Western Uganda |
| --- |

Principal Investigator(s)

| Anyolitho Maxson Kenneth |
| --- |

**Introduction**

Hello, I am a student of Mbarara University of Science and Technology (MUST) pursuing a PhD

study in development studies. It is part of a five-year project (January 2019 to December, 2023)

named: Action Towards Reducing Aquatic snail born Parasitic diseases (ATRAP) with the main

goal of this project is to aid in the prevention of snail-borne diseases in Uganda thereby lowering

the socio-economic burden created by these diseases. The project that is being implanted in the

two districts of Kagadi and Ntoroko in Western Uganda, is a collaboration between Mbarara

University of Science and Technology (MUST) and the Belgium government.

Brief background to the study

| The interview, for which you are being asked to participate in, is a study that focuses on the prevention of schistosomiasis disease in Uganda. It is hoped that the outcome of the study will greatly help in the prevention of schistosomiasis by bringing positive change in behaviors of the community. |
| --- |

**Purpose of the research project**

The purpose of this study is to examine the effectiveness of citizen science model in community

engagement to the prevention of schistosomiasis among selected rural communities of Western

Uganda. The study objectives are: to determine community knowledge, attitude and practices

regarding schistosomiasis, their health seeking behaviors, practices and attitudes, lived

experiences and perceptions regarding schistosomiasis, implementation of contextualized. The

methods that will be used to meet this purpose include In-Depth Interviews (IDIs) for some

selected members of the community that shall be deemed knowledgeable about the study topic.

The estimated duration for the sessions shall about one hour.

**Why you are being asked to participate?**

You have been asked to participate in the study on the basis that you know and understand the

importance of participating in the study and that you will provide the necessary information

required for the study.

**Procedures**

The data collection procedure shall involve your participation in in-depth interviews on knowledge, attitude, and practices including health seeking behaviours, lived experiences and perceptions regarding schistosomiasis. For proper collection of data, all sessions will be recorded both in a note book and or in an audio-recording device.

**Risks or discomforts**

There are no any known or anticipated risks associated with your participation in the study. Where such risks are known or shall be discovered in the process of the study, they shall be kept to bare minimum possible.

**Benefits**

There are no any specific benefits of this study but generally, findings of the study will help: To

improve community knowledge and attitude towards schistosomiasis; to identify and address

social and cultural factors that predispose schistosomiasis infection, to create awareness on schistosomiasis, and to scale up community involvement in schistosomiasis prevention.

**Incentives or rewards for participating**

There are no any incentives or rewards attached to this study.

**Protecting data confidentiality**

Any information shared with the research team will be treated as confidential. When processing

and publishing data, anonymity is guaranteed, as all data will be coded. This means that data that can identify a respondent will be systematically ‘translated’ into coded that will not identify the person who shared the data.

**Protecting subject privacy during data collection**

All Interviews shall be conducted in a private and quiet place chosen by the

respondent/participant. Any sensitive question that may negative affect you shall not be asked.

**Voluntarism**

Your participation in this study is voluntary, you have the right not to participate in the study if

you so wish or to leave at any moment. There is no any penalty or loss of benefits to which you

would be entitled even if you decide not to participate.

**What happens if you leave the study?**

There is no penalty that shall be levied to you if you decide to leave this study at any moment of

the session. You may withdraw from this study at any time. There is no obligation to participate

in this project.

**Compensation**

As part of expenses regarding the data collection, each participant will be given Ugx 5,000=

(Five thousand only) as compensation for the time spent while participating in the study and

transport refund where applicable of Ugx. 10,000/= (Ten thousand Shillings only), shall also be

provided.

**Who do I ask/call if I have questions or a problem?**

In case you have any questions related to the study, you may contact the chairperson of Mbarara

University of Science and Technology (MUST) Research and Ethics Committee (REC) Dr.

Francis Bajunirwe on Tel. no. 0772576396 or office Tel no. 0485433795. For any questions

related to your rights and privacy, you may contact me on Tel 0776630123, or my Supervisor Dr.

Viola Nilah Nyakato on Tel. 0772982535

**What does your signature or thumbprint on this consent form mean?**

Your signature on this form means that you have read and understood the informed consent

information which includes but is not limited to the study’s purpose, procedures, possible

benefits and risks and that you have been given the chance to ask questions before you sign. You

have also had the opportunity to ask questions about the study and any questions that you have

asked have been answered to your satisfaction and that you voluntarily consent to be a

participant in the research.

_ _ _ _ _ _ _ _ _ _ _ _ _ _ _ _ _ _ _ _ _ _ _ _ _ _ _ _ _ _ _ _ _ _ _ _ _ _ _ _ _

Name of adult participant Signature of participant or Date

Legally authorized representative

_ _ _ _ _ _ _ _ _ _ _ _ _ _ _ _ _ _ _ _ _ _ _ _ _ _ _ _ _ _ _ _ _ _ _ _ _ _ _

Name of person obtaining consent Signature Date

_ _ _ _ _ _ _ _ _ _ _ _ _ _ _ _ _ _ _ _ _ _ _ _ _ _ _ _ _ _ _ _ _ _ _ _ _ _

Print Name of witness Signature or thumbprint or mark Date
